# Facile synthesis of high strength hot-water wood extract films with oxygen-barrier performance

**DOI:** 10.1038/srep41075

**Published:** 2017-01-23

**Authors:** Ge-Gu Chen, Gen-Que Fu, Xiao-Jun Wang, Xiao-Dong Gong, Ya-Shuai Niu, Feng Peng, Chun-Li Yao, Run-Cang Sun

**Affiliations:** 1 Beijing Key Laboratory of Lignocellulosic Chemistry, College of Materials Science and Technology, Beijing Forestry University, Beijing 100083, China; 2 College of Life Science, Agricultural University of Hebei, Baoding, Hebei, 071001, China

## Abstract

Biobased nanocomposite films for food packaging with high mechanical strength and good oxygen-barrier performance were developed using a hot-water wood extract (HWE). In this work, a facile approach to produce HWE/montmorillonite (MMT) based nanocomposite films with excellent physical properties is described. The focus of this study was to determine the effects of the MMT content on the structure and mechanical properties of nanocomposites and the effects of carboxymethyl cellulose (CMC) on the physical properties of the HWE-MMT films. The experimental results suggested that the intercalation of HWE and CMC in montmorillonite could produce compact, robust films with a nacre-like structure and multifunctional characteristics. This results of this study showed that the mechanical properties of the film designated F_CMC0.05_ (91.5 MPa) were dramatically enhanced because the proportion of HWE, MMT and CMC was 1:1.5:0.05. In addition, the optimized films exhibited an oxygen permeability below 2.0 cm^3^ *μ*m/day·m^2^·kPa, as well as good thermal stability due to the small amount of CMC. These results provide a comprehensive understanding for further development of high-performance nanocomposites which are based on natural polymers (HWE) and assembled layered clays (MMT). These films offer great potential in the field of sustainable packaging.

Natureal load-bearing materials, such as seashell nacre, are well known for their extraordinary mechanical properties that result from their highly ordered brick-mortar architecture. Nacre has inspired the fabrication of synthetic layered nanocomposite materials^1^,^2^,^3^. The introduction of clay into a polymer matrix can considerably enhance the thermal and mechanical properties of the polymer film and reduce oxygen permeability^4^,^5^. Therefore, a potential route for improving the oxygen barrier and mechanical properties of polymer films is the addition of layered clays such as montmorillonite (MMT) to form polymer/clay nanocomposites. In recent years, special attention has been given to the synthesis and characterization of nanocomposites that are based on natural polymers and assembling layered clays^6^. Increased attention is being paid to renewable resources, hence the feature of hemicelluloses including their abundance, biodegradability and biocompatibility have made them attractive^7^,^8^. However, using hemicelluloses to produce materials on an industrial scale is hampered by the high production costs associated with obtaining these materials with a high degree of purity^9^. It is well known that a major fraction of wood is present in the form of hemicelluloses and lignin which are present in the waste pulping liquor and must be removed during pulping to yield undegraded cellulose fibers^10^. Thus, extracting the valuable noncellulosic components from wood chips prior to pulping and converting them into biofuels or the value-added materials is financially beneficial to the pulping industry. The purpose of the proposed hot-water extraction (autohydrolysis processing, which could lead to generation of low-molecular weight polymers) is to separate the hemicelluloses from the cellulose in the pulping process. Several researchers have found that hot-water extraction can significantly accelerate the pulping and bleaching processes^11^,^12^. However, today, the hot-water wood extract (HWE), which contains a large amount of hemicelluloses and some lignin is used primarily as a heat source, but the direct utilization of HWE in the development of new materials has not been developed. Converting the hot-water wood extract to fuel in high yield remains a much greater challenge and producing biofuels from the hot-water wood extract is still not economically viable^8^,^13^. The hot-water wood extract contains polysaccharides (mainly hemicelluloses) as a major component, a fair amount of low molecular weight lignin, and a small amount of lignin-carbohydrate complexes^14^. The lignin-carbohydrate interactions that exist in the hot-water wood extract could help to minimize gas diffusion in a polymer composite film^15^. Therefore, hot-water wood extract is an attractive material resources particularly in the food packaging industry. In addition, full use of HWE could help to reduce the consumption of chemicals and save energy, further increasing revenues to the pulping industry.

To prepare materials with improved performance, biodegradability, and environmental acceptability, one possible approach is to add layered silicate clays to produce polymer nanocomposites^16^,^17^,^18^,^19^,^20^. Layered silicate clays, such as montmorillonite (MMT), have been extensively employed as fillers or additives to reinforce polymers and fabricate nanocomposites. The layers in MMT have a thickness of around 1 nm with a mean aspect ratio of around 166^21^. MMT is a cationic clay, which consists of negatively charged layers composed of octahedral sandwiched sheets between two tetrahedral sheets. MMT can be exfoliated to separate platelets, thereby allowing the formation of film through a re-assembly process. This material also possesses a high exchange capability, as well as chemical and mechanical stability. Polymer/MMT nanocomposites have been shown to exhibit good properties, such as reduced gas permeability, lower flammability, enhanced mechanical and thermal properties^4^,^22^,^23^. Carboxymethyl cellulose (CMC) is a widely used polymer with many industrial applications, but is employed primarily used in the food chemistry^24^. CMC can be used as a filler in biocomposite film due to its high molecular weight and polymeric structure. In addition, CMC can also be used to improve the barrier and mechanical properties of composite films^25^. Consequently, in the present study, MMT and CMC were added to the hot-water wood extract to enhance its mechanical properties and obtain the balance in the network of interactions between the constituent molecules.

The goal of this work was to develop high strength, oxygen-barrier films prepared from renewable forestry product waste (hot-water wood extract). The basic concept of this effort was to generate the a layered, biomimetic composite film based on the hot-water wood extract and montmorillonite. The biomimetic nacre-like hot-water wood extract/montmorillonite (HWE-MMT) based nanocomposite films were prepared and using an industrial scalable, simple and green processing approach which was similar to the method of papermaking. The HWE-MMT based nanocomposite films, with montmorillonite as the inorganic phase, were mixed with the reinforcing agent CMC to enhance the film’s mechanical performance. The viability of the resulting products was verified based on their thermal properties, mechanical performance and oxygen permeability (OP). Another purpose of this study was to determine the optimum proportions of hot-water wood extract, MMT, and CMC to produce an ideal HWE-MMT based nanocomposite films.

## Results and Discussion

### Characterization of Nanocomposite Films

**Figure 1:**
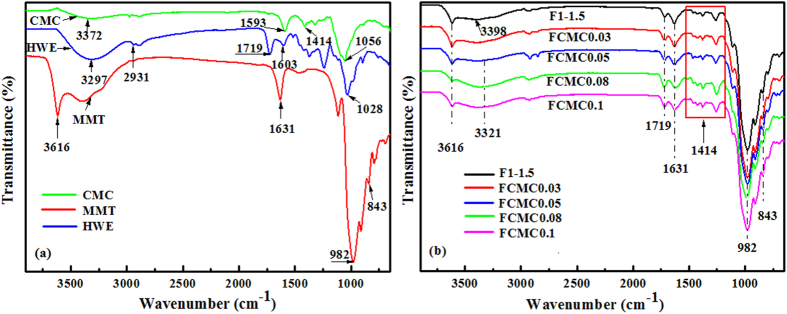
(**a**) FT-IR spectra of HWE, MMT, and CMC. (**b**) FT-IR spectra of the representative nanocomposite films (F_1-1.5_, F_CMC0.03_, F_CMC0.05_, F_CMC0.08_, F_CMC0.1_).

The FT-IR spectra of the raw materials (HWE, MMT, and CMC) are shown in Fig. 1a. The major component of the HWE is a polysaccharide fraction together with 12% lignin. The major polysaccharide component is xylan. Hence, the FT-IR spectrum of the HWE depicts the features of both hemicelluloses and lignin. The broad band at 3321 and 2931 cm^−1^ represent the –OH stretching vibrations and CH stretching. Also seen is a C=O stretching at 1719 cm^−1^ that results from the acetylated pendant groups of the polysaccharide chains. The most obvious bands at 1603, 1514, and 1425 cm^−1^ can be attributed to the aromatic regions of lignin^26^. The band at 1603 cm^−1^ is broadened by the overlaid aromatic C=C deformations, which is caused by the ring moieties in lignin. The sharp and strong band at 1028 cm^−1^ is assigned to the C-O stretching of the polysaccharide main chain. The spectrum of the pure CMC shows a broad band centered at 3372 cm^−1^ which is attributed to a wide distribution of hydrogen-bonded hydroxyl groups in the molecule. The peaks at 1593 and 1414 cm^−1^ were related to the symmetric and asymmetric modes of stretching vibration of carboxylic group (COO-)^27^. The band at 1056 cm^−1^ is attributed to the asymmetric stretching vibration band of the ether groups. In the spectrum of MMT, the frequency of the vibrational band at 3616 cm^−1^ corresponds to the -OH stretching in MMT and the band at 1631 cm^−1^ corresponds to and H-O-H bending. The peaks at 982 and 843 cm^−1^ are assigned to Si-O-Si stretching and Al (Mg)-O-H^28^,^29^,^30^.

The nanocomposite films were labeled as shown in Table 1. Compared to the FT-IR spectra of HWE, CMC, and MMT, the spectrum of the composite films for both HWE-MMT (F_1-1.5_) and HWE-MMT-CMC (F_CMC0.03_, F_CMC0.05_, F_CMC0.08_, F_CMC0.1_) exhibited the characteristic absorption of the HWE and MMT groups (Fig. 1b). The bands at 3616 and 3321 cm^−1^ corresponded to -OH stretching bond of MMT and the hydrogen-bonding of HWE. The Al-O vibrations at 843 cm^−1^ confirmed the presence of MMT, i.e., indicating that Al-O-C bonds were formed via the hydrogen-bonding interactions between the HWE and the clay platelets^28^. The FT-IR spectra of the HWE-MMT-CMC nanocomposite films with various CMC mass contents, showed a peak at around 1414 cm^−1^ which reflected the presence of the alkane groups with associated CH deformation vibration^9^. In addition, the hydroxyl group stretching of CMC appeared at 3372 cm^−1^ which was shifted to 3321 cm^−1^, this shift of hydrogen bonding was likely indicative of hydrogen-bonding interactions between HWE, CMC and MMT^15^. This change arose from a switch in the intramolecular hydroxyl-hydroxyl bonding to intermolecular hydroxyl-siloxane (Si-O-Si) hydrogen bonding^31^,^32^. The hydrogen bonds between MMT and CMC could be further strengthened forming the strong interfacial attachment needed for stress transfer^18^. The FT-IR spectra of the films also indicated that no chemical reaction occurred during film formation.

### Structure of Nanocomposite Film

**Figure 2:**
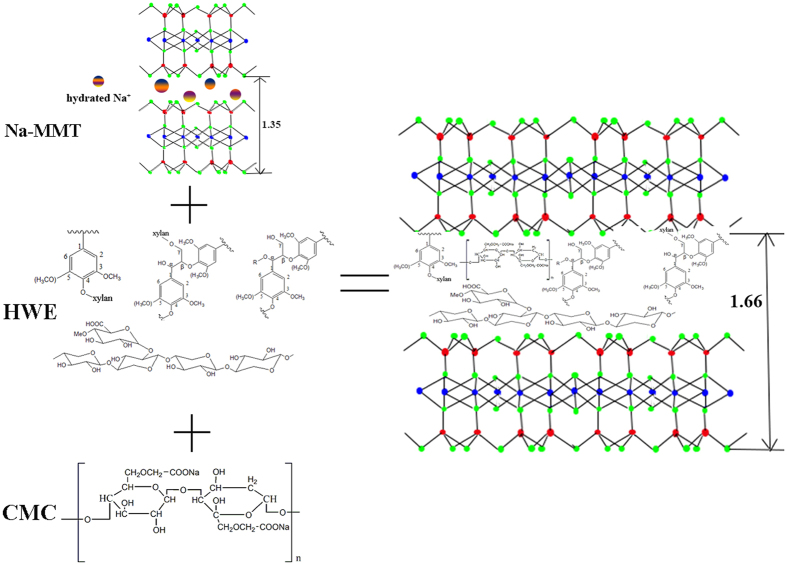
The process of HWE and CMC intercalation in MMT.

**Figure 3:**
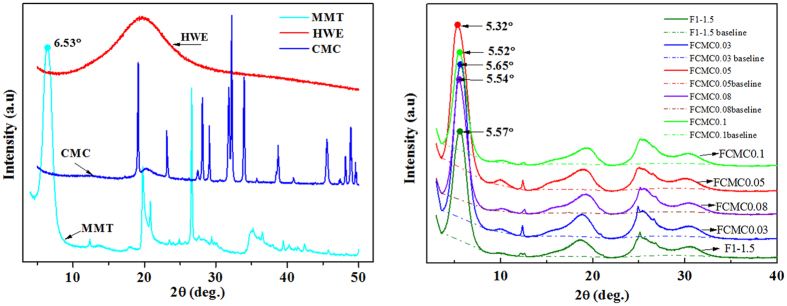
(**a**) X-ray diffraction patterns of HWE, MMT, and CMC. (**b**) The XRD patterns of the nanocomposite films F_1-1.5_, F_CMC0.03_, F_CMC0.05_, F_CMC0.08_, F_CMC0.1_.

X-ray diffraction analysis was used to evaluate if the polymer was intercalated into the MMT, and this was determined by measuring the d-spacing. The d-spacing of the MMT was calculated from the diffraction peak according to the Bragg’s equation: sin *θ* = n*λ*/2d^33^. A prominent peak for pure MMT was found at 2*θ* = 6.53°, which corresponded to the basal spacing of MMT with a *d*-spacing of 1.35 nm. In the case of HWE-MMT based nanocomposite films, this characteristic peak of MMT was shifted to ~2θ = 5.5°. In addition, the characteristic peak of the nanocomposite film F_CMC0.05_ exhibited a lower diffraction angle of 2*θ* = 5.32°, which corresponded to a *d*-spacing of 1.65 nm (Fig. 2). In other words, the interlayer distance in the MMT was increased in all XRD patterns of the HWE-MMT based nanocomposite films compared to the neat clays (Fig. 3a). This provided clear evidence of the intercalation of the polymer into the MMT. The physical properties of materials may be explained by its crystallinity as determined by the X-ray diffraction analysis. In the pattern of the HWE, a broad peak can be seen at 19.6°. The diffraction peaks at 19.1° and 32.2° were attributed to CMC. It is generally accepted that many hemicellulose based films prepared using the water casting method are semicrystalline^34^,^35^,^36^ and the X-ray diffraction results presented in Fig. 3a show that HWE exhibited no distinct crystalline peaks. Interestingly, it was found that the nanocomposite films obtained using the hot-water wood extract with MMT showed distinct crystallinity (Fig. 3b). The HWE-MMT based films (F_1-1.5_, F_CMC0.03_, F_CMC0.05_, F_CMC0.08_, F_CMC0.1_) showed distinct crystalline peaks at around 2*θ* = 18.8°, 2*θ* = 24.9°, and 2*θ* = 30.2° (Fig. 3b). This result suggested a crystalline structure in the HWE-MMT based nanocomposite films. This result also was associated with the decrease of the constant and volume of crystal lattice, and the crystal plane was changed due to the presence of the polymer (both HWE and CMC) in the MMT nanoplatelets^37^,^38^. The intensities and diffraction maxima could be used to evaluate the relative amount of crystallinity in the nanocomposite films using the software (EVA) supplied with the XRD instrument (Bruke). The crystallinities of the nanocomposite films were as follows: F_1-1.5_ 89.6%, F_CMC0.03_ 93.0%, F_CMC0.05_ 92.6%, F_CMC0.08_ 92.1%, F_CMC0.1_ 91.8%. These results indicated that the presence of CMC in the MMT impacted on the crystallinity of the HWE-MMT based nanocomposite films. The characteristic XRD peaks for MMT was shifted to lower angles and the improved crystallinity of the films may be the reason why the nanocomposite films HWE-MMT-CMC exhibited much higher tensile strength, which will be discussed in the section dealing with tensile testing.

**Figure 4:**
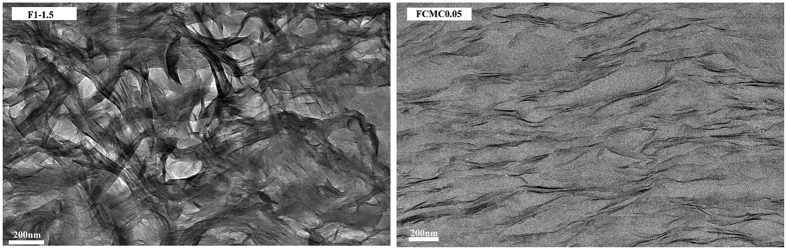
TEM micrographs of HWE-MMT based nanocomposite films: F_1-1.5_, F_CMC0.05_.

The hybrid structure of the nanocomposites was observed, and the dispersion of the nanometer-thin layered fillers in the polymer matrix was examined using TEM^39^. Hence, together with the XRD results, the dispersion of MMT platelets in HWE based films obtained from paper-making process were investigated using TEM. The TEM micrographs of the HWE-MMT nanocomposite films (F_1-1.5_ and F_CMC0.05_) are presented in Fig. 4. As the results in this Figure show, the nanolayers were well dispersed in HWE matrix with a high degree of individual layers, especially in the case of sample film F_CMC0.05_. The dark sheets of clay appeared to be more than one clay layer in the HWE matrix. In addition, no obvious association between MMT platelets could be distinguished resulting in the description of these materials for delaminated nanocomposites^40^. The results of the XRD analysis and TEM analysis suggested that the HWE-MMT based nanocomposite film were successfully prepared, and the applied paper making process lead the formation of a partially exfoliated nanocomposite structure.

### Morphology of the Nanocomposite Films

**Figure 5:**
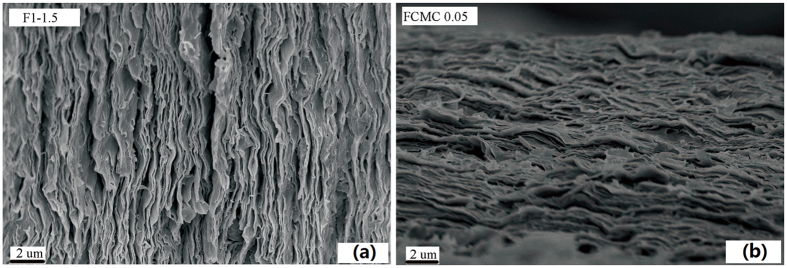
SEM images of nanocomposite films prepared from hot-water wood extract, MMT, and CMC: (**a**) F1-1.5, (**b**) FCMC0.05.

**Figure 6:**
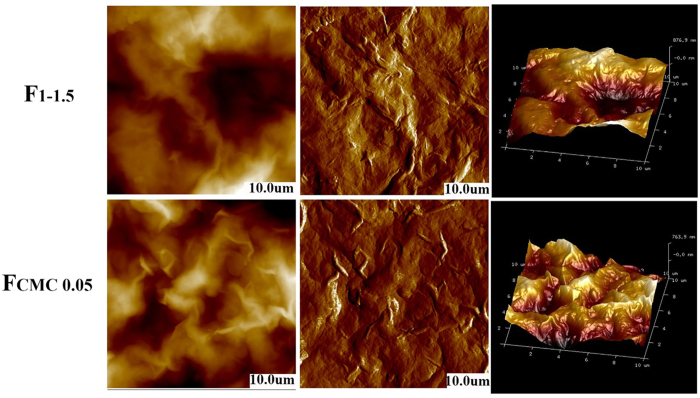
AFM images of the two nanocomposite films F_1-1.5_ and F_CMC0.05_. (The scanning scale is 10 × 10 um).

The topography of resulting films was more closely examined using SEM. Figure 5 shows the cross-sectional morphology of the nanocomposite films. The SEM images of HWE-MMT based nanocomposite films indicated that the MMT particles were well dispersed in the matrix and uniformly distributed across the cross-section. The sheet-like layers were parallel to the film surface and interpenetrated adjacent layers^16^. The cross-section of the HWE-MMT based films (F_1-1.5_ and F_CMC0.05_) were densely stacked densely together to form a well-defined layered structure, which was simiar to the brick-mortar structure of nacre. Figure 6 shows the AFM topography images of F_1-1.5_ and F_CMC0.05_. These images shows that the nanocomposite film F_1-1.5_ exhibited a rough surface morphology due to the tight intercalation of the HWE into the MMT platelets. The presence of MMT contributed to the increase in the surface roughness. This structure was also a key component in providing the film with a high gas barrier. The root-mean-square (RMS) roughness of F_1-1.5_ and F_CMC0.05_, obtained from 10 × 10 um^2^ areas of the AFM height images, were 295 and 238 nm. This indicated that a small amount of CMC made a large contribution to the surface roughness of the HWE-MMT based nanocomposite films. These result is also agree with the two AFM 3D topography images (Fig. 6). Combined with the aligned platelets and tightly packed structure, as revealed by SEM and AFM, these films were expected to provide good gas barrier performance^41^,^42^.

### Mechanical Properties of Nanocomposite Films

**Figure 7:**
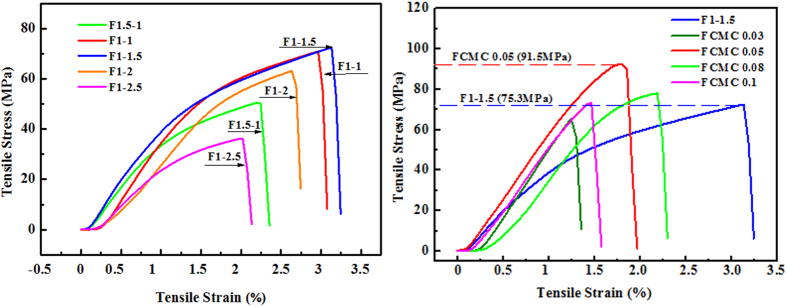
(**a**) Tensile-strain curves of the nanocomposite film HWE-MMT. (**b**) Tensile-strain curves of the nanocomposite film HWE-MMT-CMC.

To meet food packaging requirements, the HWE-MMT based nanocomposite films should have adequate mechanical performance, so all the films were be subjected to tensile testing. The resulting tensile properties showed remarkable improvements for HWE-MMT based nanocomposite films over that of the unfilled material (Fig. 7 and Table 2). In this work, the HWE films without any additives (both MMT and CMC) cannot be prepared using the water-based processing approach. The mechanical properties of the HWE-MMT nanocomposite films depended on HWE-MMT ratio. The tensile test results of the HWE-MMT nanocomposite films (F_1.5-1_, F_1-1_, F_1-1.5_, F_1-2_, F_1-2.5_), revealed that the film F_1-1.5_ exhibited a higher tensile strength than other HWE-MMT nanocomposites. The maximum tensile strength of F_1-1.5_ was 75.4 MPa with a maximum elastic deformation (*ε* = 3.3%), due to the formation of intermolecular hydrogen bonds between the HWE and MMT^43^,^44^. It was found that the optimum proportions of HWE and MMT in the nanocomposite films was 1:1.5. This result showed that the HWE-MMT based films offered superior mechanical performance in comparison to that of the films prepared using highly purified hemicelluloses and MMT^41^,^43^. This suggested that, in the HWE-MMT based nanocomposite films the higher tensile strength may be related to the native lignin and lignin-carbohydrate complexes. Based on these results, CMC was incorporated into HWE-MMT. When CMC was incorporated into the HWE-MMT matrix with an increase in the CMC ratio from 0.03 to 0.1, the maximum tensile strength of the resulting nanocomposite film HWE-MMT-CMC (F_CMC0.03_, F_CMC0.05_, F_CMC0.08_, and F_CMC0.1_) showed a higher tensile strength with a maximum tensile strength of 91.5 MPa (F_CMC0.05_). The tensile strength of the film F_CMC0.05_ was improved by approximately 21.4% compared with the film F_1-1.5_. In addition, these values were superior to those reported for films prepared using pure hemicelluloses or other polymers^40^,^43^,^44^,^45^. Compared with the literature results, the films in this study (HWE-MMT and HWE-MMT-CMC) were prepared using a small amount of raw materials, and exhibited higher relative strength. In other words, a degree of structural organization was produced by the proposed process. Another reasonable explanation for the improved tensile strength of the films in this study is that increase of CMC in HWE-MMT nanocomposite film may have improved the intercalation of HWE into MMT. In addition, the hydrogen bonding and the higher viscosity of CMC could contribute to the improved mechanical properties of the HWE-MMT-CMC films^18^. The tensile strength of the nanocomposite film F_CMC0.1_ (73.2 MPa) was lower than the film F_1-1.5_ (75.4 MPa). This indicates that excess CMC may cause structural discontinuities, which could produce polymer aggregation lowering the mechanical strength of F_CMC0.1_ compared to film (F_1-1.5_). These results revealed that a small addition of CMC can significantly improve the tensile strength the HWE-MMT based nanocomposite films. The mechanical properties of these films, in combination with XRD, SEM, AFM, and FT-IR data, demonstrated that the dispersion of MMT in polymer matrix has a special significance in improving the performance of the polymers^46^.

### Thermal Behavior Analysis

**Figure 8:**
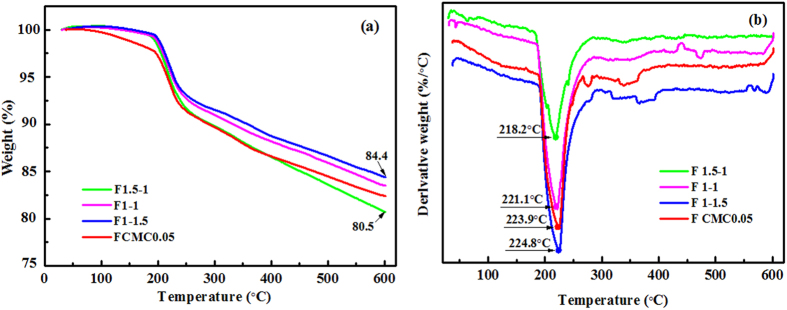
TGA (**a**) and DTG (**b**) curves of the representative films (F_1.5-1_, F_1-1_, F_1-1.5_, F_CMC0.05_).

The thermal stability of the nanocomposite films was evaluated using TG and DTG analysis and typical thermograms for the nanocomposite films HWE-MMT and HWE-MMT-CMC are presented in Fig. 8. As Fig. 8 shows, all the films exhibited two main degradation steps. The first of these was mass loss at around 180 °C where most of the water molecules desorbed. The second step in the weight loss occurred at a temperature between 200 and 600 °C, which was due to the degradation of the intercalated polymers (hot-water wood extract and CMC). Table 3 shows the weight of the residue at 600 °C in the nanocomposite films, the *T*_onset_ (the temperature at onset of the decomposition of polymer), and *T*_max_ (the maximum weight loss temperatures) were determined from the DTG curves (Fig. 8b). Evidently, the *T*_onset_ of the nanocomposite films F_1-1.5_ shifted slightly toward a higher temperature (188.4 °C) than that of the other films F_1.5-1_ (181.3 °C), F_1-1_ (186.1 °C), and F_CMC0.05_ (188.2 °C). This suggested that the *T*_onset_ of the nanocomposite films increased with MMT content (the amount of MMT in the nanocomposite films had the following order: F_1-1.5_ > F_CMC0.05_ > F_1-1_ > F_1.5-1_). The *T*_max_ of the nanocomposite films appeared to shift as a function of the MMT content, as evidence by the *T*_max_ of the nanocomposite films (F_1.5-1_ (218.2 °C), F_1-1_ (221.1 °C), F_CMC0.05_ (223.9 °C), and F_1-1.5_ (224.8 °C)). This result suggested that MMT formed a barrier in the film that hindered the diffusion of pyrolysis gases^47^. In other words, the introduction of MMT into the HWE based materials enhanced their thermal stability. This improvement in the thermal properties of the nanocomposite films can be attributed to the brick-mortar structure of films and the high thermal stability of the MMT. In addition, as can be seen from Table 3, all of the nanocomposite films exhibited a *T*_max_ at around 220 °C and the quantity of solid residues at 600 °C of the four nanocomposite films were 80.5%, 83.5%, 84.4%, and 82.4%, which implied that all the films had high thermal stability as a result of their crystal structure. The highly ordered crystal structure of the HWE-MMT based nanocomposite films also contributed to their relatively higher thermal stability. These results are in agreement with the previous XRD results.

### Oxygen Barrier Properties

A low oxygen permeability OP of food packaging materials can extend the shelf life of packaged food. The oxygen permeability of the composite films are shown in Table 4. All the films performed remarkably well as oxygen barriers with (OP) values below 2.0 cm^3^ *μ*m/m^2^·day·kPa at 0% relative humidity (RH). A material is deemed to be a good oxygen barrier if the OP is <10 cm^3^ *μ*m/m^2^·day·kPa^48^. This high OP results may be attributed to the inorganic phase (MMT) in the film, which introduced a physical barrier that increased a tortuous path for the diffusing oxygen molecules in the hot-water wood extract which eventually lengthened the characteristic length of the diffusive process^49^. As the results in the table shows, the nanocomposite film F_1-1.5_ had an OP value of 0.65 cm^3^·*μ*m/m^2^·day·kPa at 23 °C. When CMC was incorporated into the HWE-MMT matrix, the film F_CMC0.05_ also exhibited a good OP value (0.67 cm^3^·*μ*m/m^2^·day·kPa at 23 °C), which is suitable for use as a food packaging material. In addition, the OP value of the film F_CMC0.05_ (1.03 cm^3^·*μ*m/m^2^·day·kPa) was similar to film F_1-1.5_ (0.98 cm^3^·*μ*m/m^2^·day·kPa) at 40 °C. In summary, compared with previously reported films based on arabinoxylan blend of glycerol and sorbitol with an OP of 7.4 cm^3^·*μ*m/m^2^·day·kPa and 4.7 cm^3^·*μ*m/m^2^·day·kPa^50^, the HWE-MMT based nanocomposite films (both with and without CMC) exhibited good oxygen barrier properties at 23 or 40 °C. All the nanocomposite films performed far better than films composed of petroleum-based plastics such as HDPE (OP ≈ 400 cm^3^ *μ*m/day·m^2^·kPa) or PET (OP ≈ 15 cm^3^ *μ*m/day·m^2^·kPa)^51^. It is also noteworthy that our more efficient and reproducible film preparation process resulted in products with competitive OP values. These results suggest that the intercalated structure played an important role in the mechanical performance and thermal stability of the films as well as the oxygen barrier properties.

## Materials and Methods

### Materials

Hot-water wood extract (HWE) was extracted from *Populus tomentosa* wood chips, kindly supplied by Shan Dong Sun Paper Industry Joint Stock Co., Ltd. The HWE should be removed the process water using a rotary vacuum evaporator, and then freeze-dried and stored dry until use. The HWE had an average molecular weight (M*w*) of about 3348 g/mol and the polydispersity index valure (PDI) was 8.87. HWE contained mostly oligo- and polysaccharides and together with ~12% lignin. The polysaccharides were chiefly the xylans. Commercial natural clay, CloisiteNa, with a cationic exchange capacity (CEC) of 90 mequiv/100 g, was purchased from Alfa Aesar (China) Chemical Co., Ltd. Sodium carboxymethyl cellulose (CMC), with a medium viscosity of 800–1200 m Pa·s and a degree of substitution of 0.78, was obtained from Tianjin Fine Chemical Institute (Tianjin, China). Filter membrane is a microfiltration membrane with 0.22 um average pore diameter, which is made up of polyvinylidene fluoride (Jinteng, Tianjin). All reagents mentioned above were directly used without further purification.

### Nanocomposite Film Preparation

**Figure 9:**
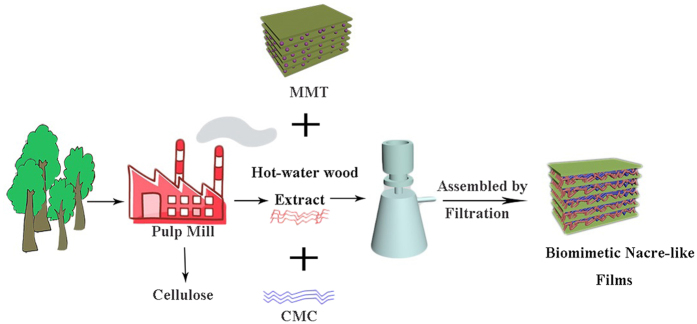
The forming mechanism of the preparation of HWE-MMT based nanocomposite films.

HWE-MMT based nanocomposite films were prepared by mixing a polysaccharide co-component (CMC), the hot-water wood extract and MMT. Firstly, solutions of each component with different concentrations were prepared separately. The solution of hot-water wood extract (5 wt%) was prepared under vigorous stirring (400 rpm) at room temperature for 2 h. A 2 wt% MMT exfoliated dispersion was achieved under magnetic stirring for 30 min, and then the suspension was sonicated using a Scientz-II D (Ningbo Scientz Biotechnoligy CO. LTD) ultrasonic processor to ensure proper exfoliation of the MMT clay, this process was repeated three times. The suspension was kept aside several days at room temperature, no sedimentation was observed and then the suspension was subsequently centrifuged to remove clay aggregates. Finally, the MMT dispersions and HWE were mixed together and stirred vigorously for 12 h at 25 °C. The ready solution (6 mL) was vacuum-filtrated with two filter membranes for 50 min and vacuum-dried at 70 °C for 10~15 min and then the nanocomposite films HWE-MMT were obtained by carefully peeling them off from the filtration membrane. In addition, CMC was selected as a co-component in HWE/MMT based film formulations (schematically illustrated in Fig. 9). Films consisting of blends of the HWE, MMT, and together with different ratios of CMC were prepared by the same method which is similar to the above process. The nanocomposite films were labeled as shown in Table 1. The forming process of the nanocomposites is illustrated in Fig. 9.

### Membrane Characterization

Attenuated total reflectance-Fourier transformed infrared (ATR-FTIR) spectra were performed to investigate the changes of functional groups in HWE/MMT based films. ATR-FTIR spectra of pure HWE, MMT, CMC, and composite films were carried out on a Nicolet 6700 spectrometer (Thermo Scientific, Pittsburgh, USA). Spectra were calculated averages of 32 scans at 4 cm^−1^ resolution from 4000 to 650 cm^−1^.

The x-ray patterns (XRD) of the nanocomposite films were carried out on a D8 advance diffractometer (Bruker, USA, Cu K*α λ* = 0.154 nm) at a generator voltage of 40 kV and a current of 35 mA. The diffraction data were collected in the range of 2*θ* = 3–50° at a scanning rate of 2° min^−1^.

The morphology of the HWE-MMT based nanocomposite films was observed by transmission electron microscopy (TEM, JEM-2010HT) operating at 200 kV. Ultrathin sections were microtomed at room temperature. Two different samples (F_1-1.5_ and F_CMC0.05_) were analyzed by TEM.

Field-emission scanning electron microscopy to assess film topography was carried out by FE-SEM (Zeiss, SIGMA) operated at 5 kV with magnifications ranging from 500 to 5000. Samples were mounted on stubs and Au coated prior to examination.

The nanomorphology of the films surface were observed by Atomic force microscopy (AFM; Bruker, Germany). Topographic (height) and phase images were recorded in tapping mode under ambient air. Root mean square (RMS) values of surface height variations were calculated across 10 × 10 um areas.

Film thickness was measured with a paper thickness gauge (ZH-4, Changchun paper testing machine CO. Ltd. China). Eight thickness measurements for mechanical properties were performed for each specimen, and the mean thickness was used to calculate the nanocomposites tensile strength.

Mechanical property of tensile strength was measured with a universal material testing machine equipped with a 100 N loading cell. Each film was cut into10 mm width and a length of 40 mm. The samples were conditioned and tested at room temperature and 50% relative humidity (RH). The sample was placed between the testing plates with a gauge length of 20 mm, and the test was run at a pulling speed of 5 mm/min. Sample results was averaged over 6–8 specimens.

Thermogravimetric analyses (TGA) and derivative thermogravimetry (DTG) were completed on a simultaneous thermal analyzer (DTG-60, Shimadzu, Japan). TGA tests were conducted and samples were heated from 40 °C to 600 °C with a heating rate of 20 °C/min.

The oxygen barrier properties of the HWE/MMT based nanocomposite films were performed with a VAC-V1 permeability analyzer with a carrier flow (N_2_) of 10 mL/min in accordance with the standard method GB/T 1038–2000. The oxygen permeability determinations were done on a 4.9 cm^2^ surface sample and the partial pressure of oxygen was 1 atm. The OP of films were reported in cm^3^ *μ*m/day·m^2^·kPa at 23 and 40 °C, the relative humidity (RH) condition was 0%.

## Conclusions

The reported work was the first attempt to introduce MMT into a hot-water wood extract matrix to produce nanocomposite films with a brick-mortar structure. A proper balance of the network of interactions between the constituent molecules was further advanced by addition of the reinforcing agent CMC. A robust and environmentally friendly method similar to paper-making was adopted for the preparation of the hot-water wood extract/MMT based nanocomposite films which exhibited good heat-resistance, excellent gas barrier properties, and high strength. The self-assembly behavior in this HWE/MMT hybrid system was confirmed by XRD analysis, and the HWE-MMT based nanocomposite films showed distinct crystallinity. In addition, the presence of CMC made improved to the crystallinity of the HWE-MMT nanocomposite film (F_CMC0.03_ 93.0%, F_CMC0.05_ 92.6%). Compared to the HWE-MMT film, the HWE-MMT-CMC films exhibited excellent tensile strength (91.5 MPa) as a result of the small amount of CMC. The tensile strength of the composite films was 21.4% higher than the nanocomposite film F_1-1.5_. This result test confirmed that a small amount of CMC can enhance the mechanical performance of the HWE-MMT based nanocomposite films. In other words, the mechanical properties of HWE-MMT based nanocomposite films were better than the films composed of highly purified hemicellulose. In addition, the HWE-MMT-CMC nanocomposite films also had exhibited good thermal behavior and lower oxygen permeability (<2.0 cm^3^ *μ*m/day·m^2^·kPa). In summary, converting hot-water wood extract into value-added materials (films) can lower the production cost, covert waste material into a value added product and also benefit the environment. This study provides a new concept for the utilizing hot-water wood extract from biorefineries and pulping mills. The results showed that the introduction of CMC into the HWE-MMT films played an important role in the resulting of mechanical property, oxygen permeability, and thermal stability of the film product. These results suggest that the HWE-MMT-CMC nanocomposite films may offer great potential in the field of packaging materials, by increasing the competitiveness in terms of the environmental foot print, performance, and cost of the product.

## Additional Information

**How to cite this article:** Chen, G.-G. *et al*. Facile synthesis of high strength hot-water wood extract films with oxygen-barrier performance. *Sci. Rep.* **7**, 41075; doi: 10.1038/srep41075 (2017).

**Publisher's note:** Springer Nature remains neutral with regard to jurisdictional claims in published maps and institutional affiliations.

## Figures and Tables

**Table 1:** Films with different proportions in volume of HWE, MMT, and CMC.

Sample Code	Proportion^*α*^
F_1.5-1_	V(HWE)/V(MMT) = 1.5:1
F_1-1_	V(HWE)/V(MMT) = 1:1
F_1-1.5_	V(HWE)/V(MMT) = 1:1.5
F_1-2_	V(HWE)/V(MMT) = 1:2
F_1-2.5_	V(HWE)/V(MMT) = 1:2.5
F_CMC0.03_	V(HWE)/V(MMT)/V(CMC) = 1:1.5:0.03
F_CMC0.05_	V(HWE)/V(MMT)/V(CMC) = 1:1.5:0.05
F_CMC0.08_	V(HWE)/V(MMT)/V(CMC) = 1:1.5:0.08
F_CMC0.1_	V(HWE)/V(MMT)/V(CMC) = 1:1.5:0.1

^α^The concentration of HWE was 5 wt%, and the concentrations of MMT and CMC were 2 wt%.

**Table 2:** Tensile testing results of the nanocomposite films.

Samples	Tensile strength (MPa)		Tensile strain (%)	Thickness (um)	
F_1.5-1_	53.5 ± 6.3		2.1 ± 0.9	42.7	
F_1-1_	73.6 ± 7.1		2.9 ± 0.5	48.1	
F_1-1.5_	75.4 ± 5.6		3.3 ± 0.2	33.8	
F_1-2_	62.2 ± 4.7		2.7 ± 0.8	35.4	
F_1-2.5_	39.7 ± 4.3		1.9 ± 0.4	36.3	
F_CMC0.03_	60.7 ± 6.9		1.8 ± 0.5	35.9	
F_CMC0.05_	91.5 ± 4.0		2.1 ± 0.2	36.0	
F_CMC0.08_	77.7 ± 8.2		2.2 ± 0.3	36.1	
F_CMC0.1_	73.2 ± 3.4		1.7 ± 0.4	36.7	
**Films reported in literatures**					
**Reference**	**Major component**	**Additional components**	**Thickness (um)**	**Tensile strength (MPa)**	**Tensile strain (%)**
43	Quaternized hemicelluloses	MMT	49–62	19.3–57.8	2.0–3.2
44	Wood hydrolysate	MMT/Talc	100–200	61.0–77.6	2.1–4.7
45	Banana flour	MMT	180	23.4–42.9	8.3–11.6

**Table 3:** Thermal properties of nanocomposites.

Curve	T_*onset*_ (°C)	T_max_ (°C)	Residuals (wt%) at 700 °C
F_1.5-1_	181.3	218.2	80.5
F_1-1_	186.1	221.1	83.5
F_1-1.5_	188.4	224.8	84.4
F_CMC0.05_	188.2	223.9	82.4

**Table 4:** Oxygen Permeability (cm^3^ *μ*m/day·m^2^·kPa) of the nanocomposite films.

Sample	Thickness (um)	23 °C 0% RH	40 °C 0% RH
F_1-1.5_	33.8	0.65	0.98
F_CMC0.05_	36.0	0.67	1.03
